# In vivo imaging reveals mature Oligodendrocyte division in adult Zebrafish

**DOI:** 10.1186/s13619-021-00079-3

**Published:** 2021-06-02

**Authors:** Suqi Zou, Bing Hu

**Affiliations:** 1 Institute of Life Science, Nanchang University, Nanchang, Jiangxi 330031 P. R. China; 2 School of Life Sciences, Nanchang University, Nanchang, Jiangxi 330031 P. R. China; 3 Hefei National Laboratory for Physical Sciences at the Microscale, University of Science and Technology of China, Hefei, Anhui 230026 P. R. China

**Keywords:** Remyelination, Mature oligodendrocyte, Zebrafish, In vivo imaging, Asymmetric division

## Abstract

Whether mature oligodendrocytes (mOLs) participate in remyelination has been disputed for several decades. Recently, some studies have shown that mOLs participate in remyelination by producing new sheaths. However, whether mOLs can produce new oligodendrocytes by asymmetric division has not been proven. Zebrafish is a perfect model to research remyelination compared to other species. In this study, optic nerve crushing did not induce local mOLs death. After optic nerve transplantation from olig2:eGFP fish to AB/WT fish, olig2^+^ cells from the donor settled and rewrapped axons in the recipient. After identifying these rewrapping olig2^+^ cells as mOLs at 3 months posttransplantation, in vivo imaging showed that olig2^+^ cells proliferated. Additionally, in vivo imaging of new olig2^+^ cell division from mOLs was also captured within the retina. Finally, fine visual function was renewed after the remyelination program was completed. In conclusion, our in vivo imaging results showed that new olig2^+^ cells were born from mOLs by asymmetric division in adult zebrafish, which highlights the role of mOLs in the progression of remyelination in the mammalian CNS.

**Supplementary Information:**

The online version contains supplementary material available at 10.1186/s13619-021-00079-3.

## Background

Multiple sclerosis (MS), a chronic autoimmune disease, is histologically characterized by the formation of demyelination and remyelination plaques in the central nervous system (CNS) (Tanaka and Yoshida 2014). A prerequisite to devising effective remyelination-enhancing therapies is a detailed understanding of the molecular and cellular mechanisms of how remyelination occurs (Franklin et al. 2020). However, the source of myelin-forming cells in remyelination has been a central theme for several decades (Franklin and Ffrench-Constant 2008). An accepted dogma is that remyelination in the adult animal is the recapitulation of myelination occurring in development, in which oligodendrocyte progenitor cells (OPCs) are the major source of remyelinating cells. This hypothesis supports that OPCs account for 5–8% of all cells in the adult brain (Franklin and Ffrench-Constant 2008)**,** that OPCs appear before myelin recovery in the plaque areas of MS patients (Scolding et al. 1998), and that transplantation of OPCs to the adult brain facilitates remyelination and functional restoration (Groves et al. 1993). In vivo research in transgenic mice supports the hypothesis that NG2-positive OPCs can proliferate and maintain the balance of OPCs (Hughes et al. 2013).

However, recent research has reinvigorated the theory that mOLs mainly contribute to remyelination (Duncan et al. 2018). Transplantation-sorted mOLs were more capable of generating myelinated oligodendrocytes in vitro and in vivo (Duncan et al. 1992; Wood and Bunge 1991). In the EAE model induced by recombinant MOG in adult mice, mOLs remained intact, though their myelin was stripped (Romanelli et al. 2016). In the focal cerebral ischemia model (McIver et al. 2010) and the spinal cord demyelination model (Jeffries et al. 2016), mOLs survived and regrew processes into the lesion areas. The results from two large animal demyelination and remyelination models, in which cats were fed irradiated food and rhesus monkeys were fed vitamin B12-deprived food, also support this opinion that surviving mOLs could participate in remyelination by extending new processes to ensheath demyelinated axons (Duncan et al. 2018). Recently, according to a mathematical model that analyzed the nuclear bomb test-derived ^14^C level in oligodendrocyte nuclei of the human brain, myelin exchange occur at a high rate, while the oligodendrocyte population is remarkably stable in white matter after 5 years of age. They concluded that mOLs contribute mainly to myelin modulation in human white matter, which facilitates rapid neural plasticity in the development period (Yeung et al. 2014). Subsequently, the group also assessed oligodendrocyte generation in multiple sclerosis patients and found that most patients failed to generate new oligodendrocytes in normal-appearing white matter and that newly produced oligodendrocytes were minimally present in shadow plaques (Yeung et al. 2019). Their unexpected results implied that remyelination in humans is carried out by old spared oligodendrocytes rather than by new oligodendrocytes (Yeung et al. 2019). These studies provide an alternative model in which surviving compromised oligodendrocytes produce new myelin sheaths to participate in remyelination in the areas of MS-induced plaques (Franklin and Ffrench-Constant 2008).

However, another possible pathway depends on whether mOL proliferation during remyelination is believed impossible, although such was implied in 1984 (Ludwin 1984). Considering that mOLs are completely differentiated cells and have a weak ability to migrate in the CNS, most people believe that mOL division during remyelination is negligible (Crawford et al. 2016). Although in vitro studies show that mOLs can re-enter the cell cycle when stimulated by extra factors (Fressinaud et al. 1993), research also indicates that oligodendrocytes can mitose with intact process integrity in vivo (Arenella and Herndon 1984; Ludwin 1984; Sturrock and McRae 1980). An example of completely differentiated cells maintaining proliferation ability is Müller cells in the retina in low vertebrate animals. These cells play the role of stem cells to produce all kinds of cells within the retina through an asymmetric division pathway, characterized as a status in which nuclear reprogramming is complete with process integrity (Lahne and Hyde 2017). Do mOLs participate in the progression of remyelination by cell division?

To determine whether mOLs participate in remyelination, we focused on mOL proliferation with real-time in vivo imaging in adult zebrafish. Here, taking advantage of transgenic lines (Bin and Lyons 2016) and the optic nerve remyelination model of adult zebrafish (Zou et al. 2013), we found that local mOLs survived in the optic nerve crush model and proliferated when they were mature at 3 months after transplantation. In addition, under stimulation with inflammatory factors, asymmetrical mOL division within the retina was also captured by in vivo imaging. Our results provide the first direct evidence that mOLs can indeed divide in vivo, which will arouse our interest in revealing the role of mOLs in the remyelination of the mammalian CNS.

## Results

1. Oligodendrocytes in the distal segment survive and proliferate after optic nerve injury

**Fig. 1:**
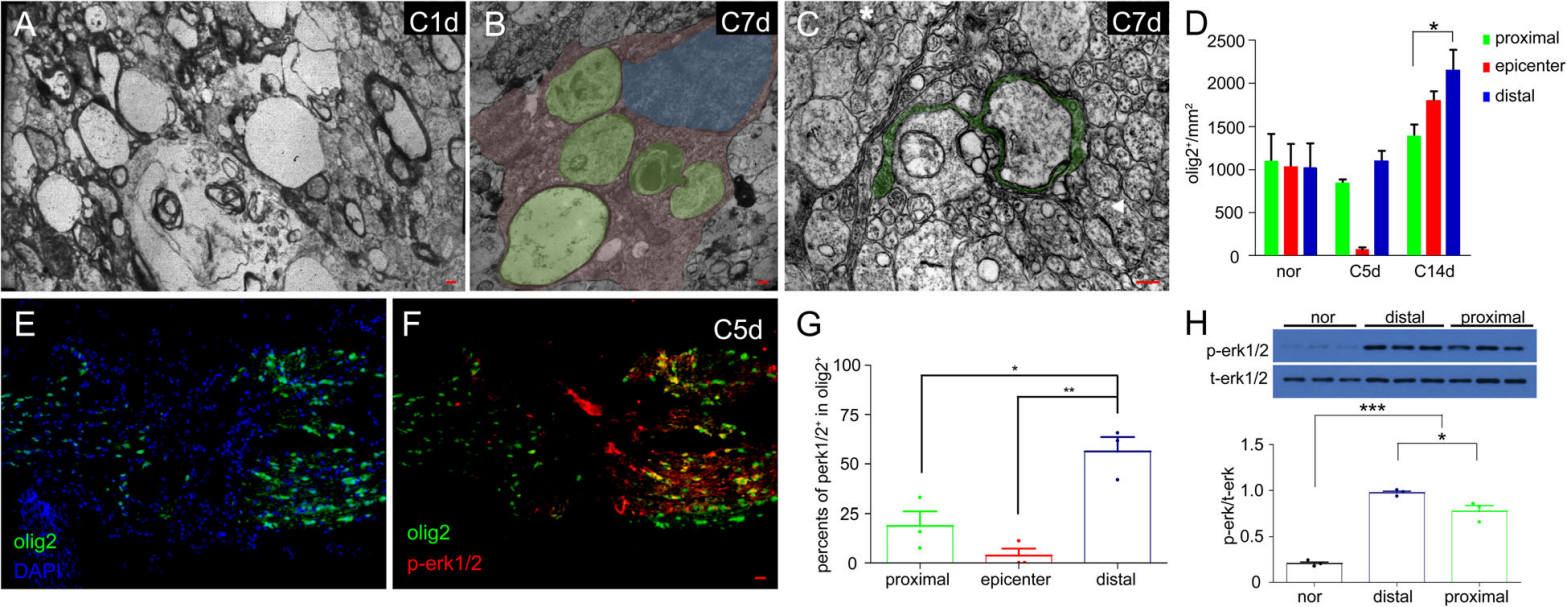
Oligodendrocytes survived after optic nerve injury. **a** At 1 dpi, axons degenerated in the distal stump, inducing myelin collapse. **b** At 7 dpi, microglia/macrophages (pink) ingested myelin debris (green). **c** Remyelination (green) started to ensheath regenerated axons at 7 dpi. **d** Statistical analysis of olig2^+^ cells in the proximal segment, epicenter, and distal segment at different times after injury. **e**-**f** At 5 dpi, olig2^+^ cells were not found in the epicenter. Most olig2^+^ cells in the distal stump (right side) expressed p-erk1/2 (yellow cells), while the proximal cells (left side) did not. **g** The percentage of p-erk1/2 in olig2^+^ cells in the distal segments was significantly higher than that in the proximal segments at 5 dpi. **h** Compared with the expression level of p-erk1/2 protein in the normal optic nerve, the proximal segments and the distal segments were at 5 dpi by western. Scale bar: 200 nm (**a**-**c**), 20 μm (**f**).

To investigate the fate of oligodendrocytes after optic nerve crushing, we observed myelin collapse in the distal stump on the first day after injury (Fig. 1a) and found that myelin debris was swallowed by invading microglia/macrophages at 7 dpi (Fig. 1b). Interestingly, the start of remyelination was also observed at 7 dpi (pseudocolor in Fig. 1c), which suggested that oligodendrocytes in the distal segments would survive after optic nerve injury. Next, we counted the number of olig2^+^ cells in the optic nerve (Fig. 1d). The results showed that oligodendrocytes were lost in the epicenter before 7 dpi but re-entered at 14 dpi (Munzel et al. 2012). In the distal stump, olig2^+^ cells existed during the whole period. At 14 dpi, the olig2^+^ cell number in the distal region was greater than that in the proximal region, which implied that oligodendrocytes proliferated in this stage (Fig. 1d). As p-erk1/2 is regarded as a specific marker for oligodendrocyte survival and proliferation (Hu et al. 2008; Lin et al. 2013), we detected its expression in the optic nerve and found that most olig2^+^ cells in the distal segment were p-erk1/2 positive at 5 dpi (Fig. 1e-f). Statistical results showed that the ratio of p-erk1/2^+^ to olig2^+^ cells in the distal stump was larger than that in the proximal segment (Fig. 1g). Western blot analysis also indicated that the expression of p-erk1/2 in the distal stump was significantly higher than that in the proximal stump at 5 dpi (Fig. 1h). All these results indicated that oligodendrocytes survived and proliferated after optic nerve injury in adult zebrafish.

2. **mOLs from the donor optic nerve participate in remyelination in host fish**

**Fig. 2:**
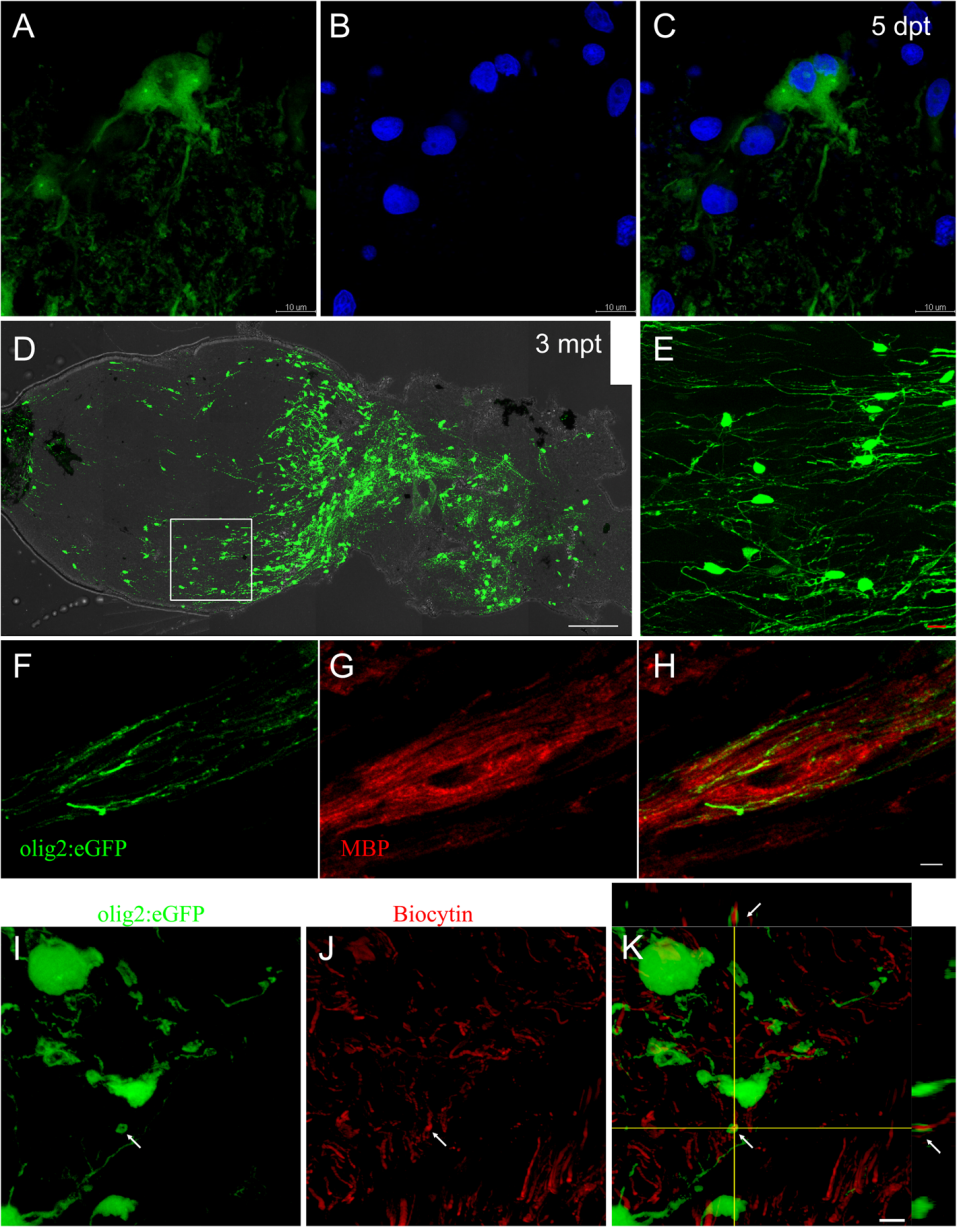
mOLs from donor adult zebrafish rewrapped axons in the host fish at 3 mpt. **a**-**c** Two nuclei were found in an olig2^+^ cell at 5 dpt (single focus), which suggests that olig2^+^ cells could proliferate in the host zebrafish. **d**-**e** Olig2^+^ cells from the donor optic nerve survived in the host (**d**) and remyelinated along the whole optic nerve (**e**). **f**-**h** MBP staining showing that olig2^+^ processes were mature myelin processes (single focus). **i**-**k** In the cross-section, olig2^+^ cells in the host fish rewrapped regenerated axons (arrows). Scale bars: 10 μm (**a**-**c**, **e**), 100 μm (**d**), 5 μm (**f**-**h**); 2 μm (**i**-**k**). The left side in (**d**-**h**) represents the proximal segment

To investigate whether local mOLs could be a resource of newly formed oligodendrocytes, we transplanted optic nerve segments from adult olig2:eGFP fish into the gap of adult AB/WT optic nerves (Tian et al. 2016). As Fig. 2a-c shows, some olig2^+^ cells in the host optic nerve had generated two nuclei at 5 days posttransplantation (dpt). At 3 months posttransplantation (mpt), olig2^+^ cells had scattered along the whole optic nerve in the host (Fig. 2d) and remained in a state similar to mOLs (Fig. 2e). To identify whether these olig2^+^ cells were mOLs, we performed MBP staining and found that these olig2^+^ processes were colocalized with MBP (Fig. 2f-h). Additionaly, RGC axons were ensheathed by olig2^+^ processes in the longitudinal section, as shown in Fig. S1A-C. In the cross-section, olig2^+^ myelin processes were observed around biotin-labeled regenerated axons (Fig. 2i-k). Under immunoelectron microscopy, eGFP^+^ processes in normal olig2:eGFP fish were specifically located in the myelin sites; we also detected some eGFP^+^ processes around the regenerated axons in the host fish at 3 mpt (Fig. S1D-E). These results indicated that local mOLs from the adult zebrafish optic nerve could survive and participate in remyelination progression.

3. **In vivo imaging detects the proliferation of local oligodendrocytes in the optic nerve**

**Fig. 3:**
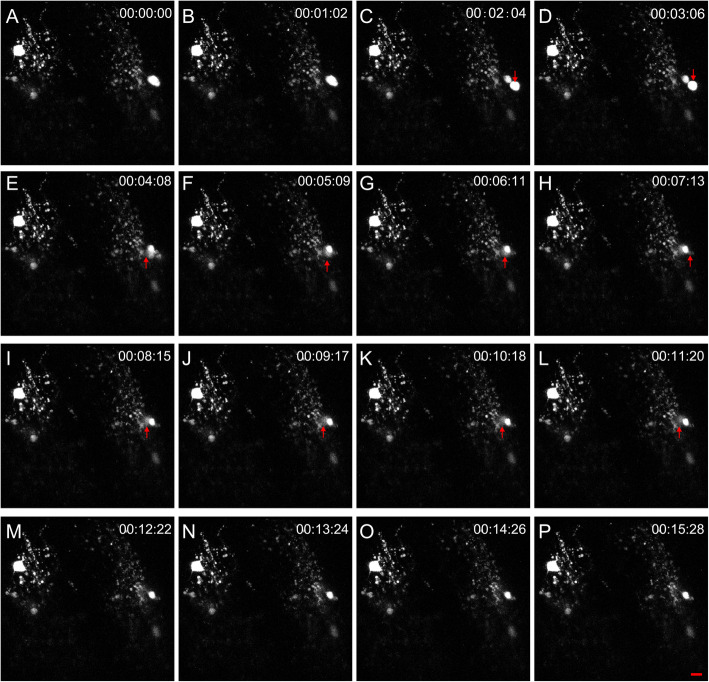
Imaging proliferation of local oligodendrocytes in the transplanted optic nerve. Two olig2^+^ cells in the transplanted optic nerve project their processes to large areas, suggesting that they are complex oligodendrocytes. At 1 min 2 s, olig2^+^ cell division was not observed. At 2 min 4 s, a new olig2^+^ cell was divided from one olig2^+^ cell, and the whole progress was completed within 1 min. The newborn olig2^+^ cell migrated to the right at 3 min 6 s (descending arrow) and then migrated into the deeper space from 4 min 8 s to 11 min 20 s (ascending arrow). Finally, the cell migrated out of focus at 12 min 22 s. Each image has 25 optic slices, the interval is 1 μm, and the scale bar is 10 μm

How do transplanted mOLs participate in remyelination? Do they differentiate into OPCs or produce new OPCs directly? To answer these questions, we observed local mOL division in the transplanted optic nerve in vivo, taking advantage of olig2^+^ cells sporadically distributed along the host optic nerve at 3 mpt (Fig. 2d-e). As shown in Fig. 3, there were only two olig2^+^ cells in the first frame of the imaging field. According to the complex morphology of oligodendrocytes with all their processes parallel to the optic nerve, we believe these cells are mOLs similar to those in Fig. 2d-k. In the third frame (Fig. 3c), an ecptoma was suddenly dissected from the soma of the right olig2^+^ cell, after which it migrated into deeper space (from the fifth frame to the twelfth frame) and finally out of imaging focus at 12 min 22 s (Movie S1). As shown in Fig. S2, we obtained more details of olig2^+^ cell division in the same animal. Before cell division, the fluorescence of the soma was concentrated, and the myelin process was kept intact. In the fourth frame, some cytoplasm with weak fluorescence crawled under the focus. Three minutes later (i.e., the fifth frame to the seventh frame), the lower part of the cytoplasm migrated to the left side and formed a small appendage attached to the soma (arrowheads in the seventh frame to the tenth frame). The appendage kept moving to the left and then was completely divided from the soma in the ninth frame (Fig. S2, Movie S2). After transplanting olig2^+^ cells that can rewrap regenerated axons, our in vivo imaging results confirmed that local oligodendrocytes in the optic nerve could directly participate in remyelination progression.

4. **In vivo imaging shows that mOLs within the retina generate new OPCs in an asymmetric manner**

**Fig. 4:**
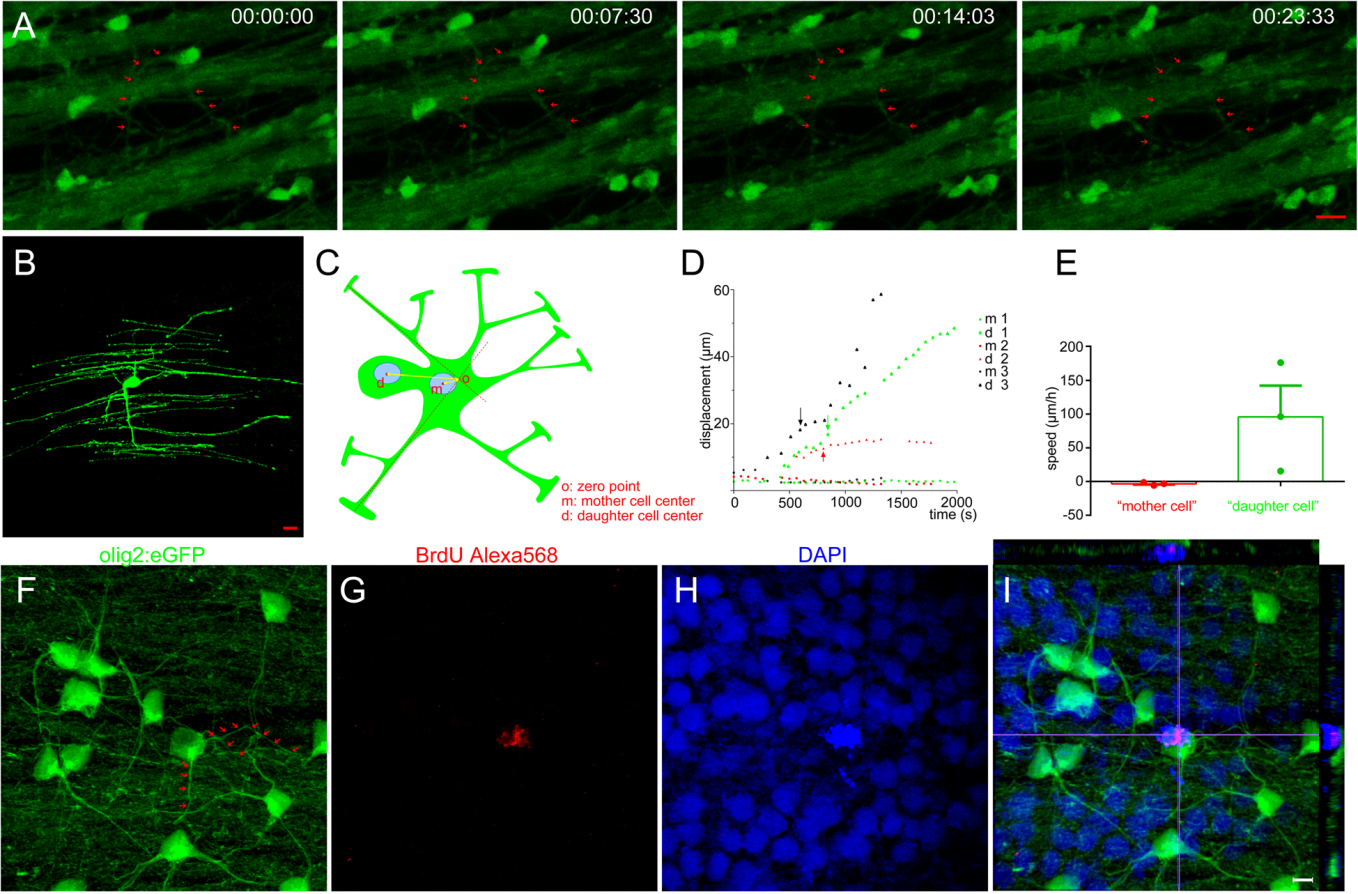
mOLs within the retina are divided in an asymmetric manner. **a** Serial imaging showed that the mOL within the retina was under division. Arrows indicate the processes that maintained integrity during the whole imaging. **b** The olig2^+^ cell within the retina is in a mature state, as shown by the perfect myelin structure. **c** Schematic picture shows the method to calculate cell displacement in the asymmetric division of mOL. The origin point is the crossover of two random myelin processes. **d** The displacement of the mother and daughter cells in the asymmetric division of mOLs within the retina. Arrows show the time point at which division was completed. **e** Speeds of mother and daughter cells. Daughter cells had a high migration ability while mother cells did not (*p* < 0.05). **f**-**i** BrdU results show that a mOL within the retina was proliferating, while the myelin structure remained integral (arrows). The image has 10 slices, and the interval is 0.5 μm. Scale bar: 10 μm (**a**-**b**); 5 μm (**f**-**i**)

Here, aiming to obtain higher-quality imaging of mOL division in vivo, we focused on oligodendrocytes within the retina (Fig. S3B). Our previous results showed that the myelin sheath in the retina was colocalized with MBP antibody and had Ranvier node structure, meaning that there are mOLs present in the adult zebrafish retina (Tian et al. 2016; Zou et al. 2013). Additionally, Fig. 4b shows that an olig2^+^ cell within the retina displayed a perfect mOL morphology. To ensure a high chance of imaging mOL division in vivo, we injected 5 μg/ml TNFα solutions into the normal eyes of olig2:eGFP fish every 4 h (Arnett et al. 2001; Cunha et al. 2020). After the third injection, we successfully observed mOL division in vivo (2/12 fishes). As indicated by the arrows in Fig. 4a, the processes of mOLs maintained their integrity during the whole imaging period. At 7.5 min, a spore emerged from the soma, and the division was completed in 14 min 3 s. At 23 min 33 s, the newborn olig2^+^ cell moved 27.4 μm from the mother cell (Fig. 4a, Movie S3). As the daughter cells were directly and asymmetrically divided from the soma of the mother cells, we calculated the displacement between the center of the mother cell or the daughter cell and the reference point (the cross-site of two myelin processes, Fig. 4c). The results showed that the displacement of the daughter cells was significantly larger than that of the mother cells (Fig. 4d). In Fig. 4e, the mean speed of the daughter cells was 95.9 ± 46.3 μm/h (*n* = 3) in the first half-hour, which was significantly higher than that of the mother cells (− 3.48 ± 1.25 μm/h, n = 3). To confirm whewther mOLs within adult zebrafish could directly produce new cells in an asymmetric manner, we administered BrdU to label those mOLs under division. As shown in Fig. 4f, the central olig2^+^ cell was mOL, as it projected several processes (arrows). The nucleus (Fig. 4h) of this mOL has incorporated BrdU (Fig. 4g). The orthogonal image shown in Fig. 4i has an imaging Z stack of only 6 μm, confirming that the mOL incorporated BrdU. To our knowledge, this was the first study to capture mOL division in adult vertebrates in vivo.

5. **The Myelin structure recovers completely in the optic nerve crush model**

**Fig. 5:**
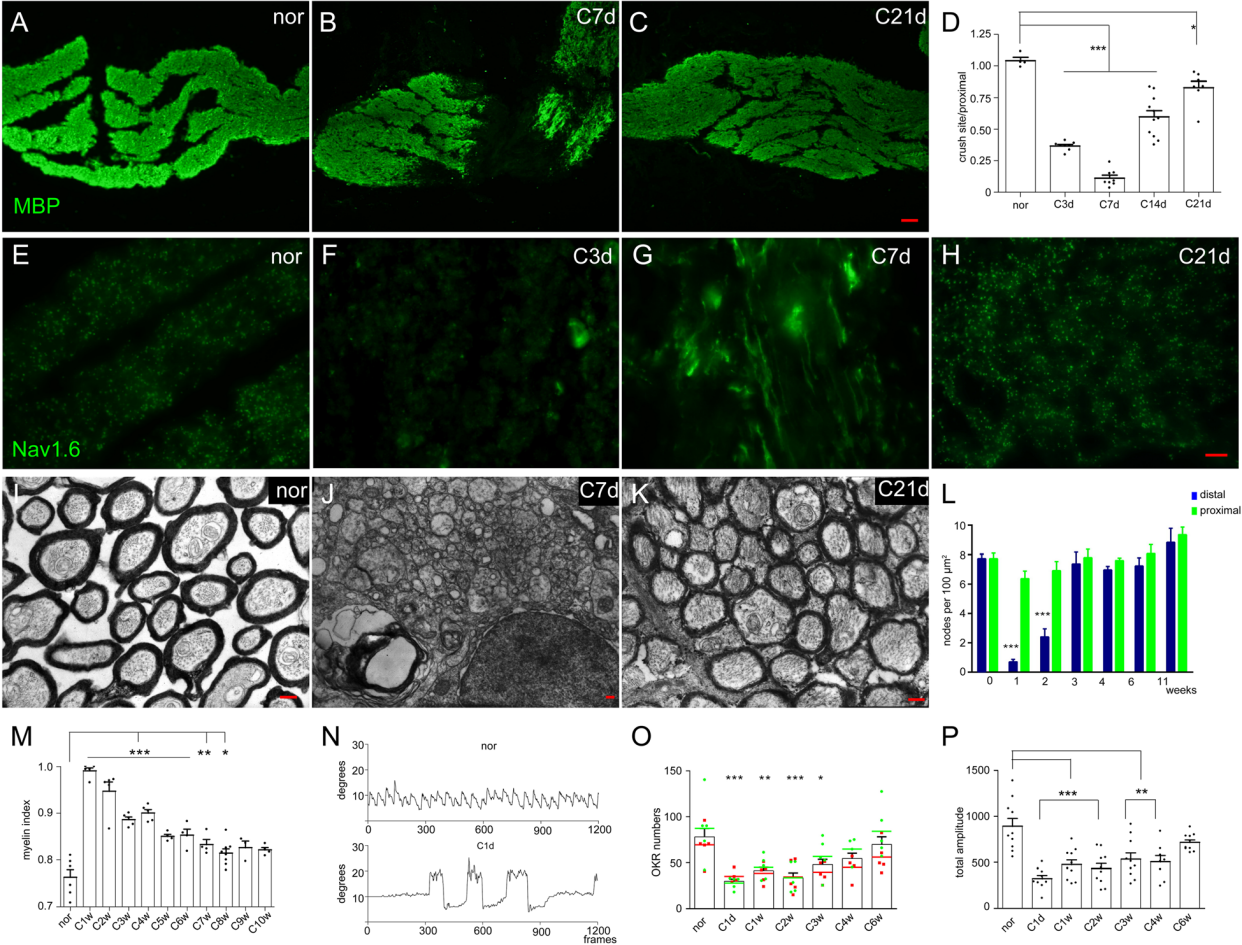
Visual function recovery after the completion of remyelination. **a**-**c** Qualitative results showing the restoration of MBP at different times after optic nerve injury. **d** Statistical results showing that the MBP intensity at the injury site was lowest at 7 dpi and gradually recovered in the following weeks. **e**-**h** Qualitative images showing Nav1.6 staining at different times after injury. At 7 dpi, Nav1.6 in the distal stump presented a linear pattern (**g**). **i**-**k** Qualitative images showing the myelin sheath at different times after optic nerve injury. Demyelination was completed at 7 dpi (**j**), but remyelination mostly occurred at 21 dpi (**k**). **l** Statistical results showing that the density of Ranvier nodes in the proximal region was not influenced by optic nerve injury, while it was greatly decreased in the distal region during the first two weeks and then returned to normal at 3 wpi. **m** Statistical results showing that the myelin index was gradually restored in the following weeks and completely finished at 9 wpi (*p* > 0.05). **n** Qualitative figures showing the nystagmus of normal and injured fish in the ocellanae OKR test. On the first day after optic nerve injury (down line), eye movement was voluntary (the large amplitude movements), and the nystagmus was much weaker in injuried fish than in normal fish (up line). **o** The preference of clockwise orientation in the injured eye was recovered at 3 wpi, and the OKR number was recovered at 4 wpi, indicated that the second-order visual function was restored. **p** The total amplitude of right eye nystagmus recovered, and the results indicated that fine visual function was restored at 6 wpi. The left side in A-C represents the proximal segment. Scale bar: 20 μm (**a**-**c**); 10 μm (**e**-**h**); 200 nm (**i**-**k**)

The results described above showed that new OPCs could be generated from local mOLs. Here, to identify when remyelination is completed after optic nerve injury in adult zebrafish, we checked the myelin structure by immunohistochemistry and TEM. Figure 5a-c shows that the MBP signal in the epicenter disappeared at 7 dpi and reemerged at 21 dpi (Munzel et al. 2012). The ratio of the MBP intensity of the crush site to that of the proximal stump was significantly decreased before 14 dpi, slightly recovered at 21 dpi and reached a normal level at 28 dpi (Fig. 5d). We labeled sodium channels with a nav1.6 antibody to research Ranvier node recovery after optic nerve injury (Waxman 2006). As Fig. 5e shows, Ranvier nodes were intensively scattered in the optic fascicle of the normal optic nerve. At the early stage of optic nerve injury, Ranvier nodes disappeared as axons degenerated (Fig. 5f). At 7 dpi, some nav1.6 linear patterns were seen in the distal segments, which suggested that these regenerated axons had not been sheathed by oligodendrocytes (Fig. 5g). At 3 wpi (weeks postinjury), most Ranvier nodes had recovered (Fig. 5h), and the statistical results showed that there was no significant difference from the normal group (Fig. 5l). Under TEM observation, we analyzed the myelin thickness at different times after injury. In the normal optic nerve (Fig. 5i), the myelin around axons was compact. At 7 dpi, when demyelination had been completed, most regenerated axons were naked (Fig. 5j). However, at 21 dpi, the myelin sheath of regenerated axons was restored gradually (Fig. 5k). Myelin index results showed that the myelin thickness was completely recovered at 9 wpi (Fig. 5m; Fig. S4C).

To test the fine visual function recovery of experimental eyes, we performed an OKR test in adult zebrafish with ocellanae injury. In Fig. 5n, the upper line shows the nystagmus of a normal fish, and the bottom line shows the nystagmus of an injured fish at 1 dpi. The nystagmus number and amplitude significantly decreased after optic nerve injury. The nystagmus number showed that clockwise dominance was restored at 3 wpi, and the frequency recovered at 4 wpi (Fig. 5o). By analyzing the total nystagmus amplitude each week after optic injury (Fig. 5p), we found that fine visual function was completely restored at 6 wpi (*p* > 0.05) when remyelination was completed. All these results showed that zebrafish recovered not only coarse and fine visual function but also the second-order visual function after remyelination was completed (Orger et al. 2008).

## Discussion

Whether mOLs could participate in the remyelination progress has been intensely contended over several decades (Franklin and Ffrench-Constant 2008). We used the optic nerve crush model in adult zebrafish to confirm that mOLs in the distal stump survived and proliferated after injury. Then, we transplanted a segment of the optic nerve from adult olig2:eGFP to adult AB/WT fish and found that mature olig2:eGFP oligodendrocytes could ensheath regenerated axons in the host optic nerve. Our in vivo imaging results directly revealed that local mOLs in the optic nerve and within the retina could produce new OPCs from the soma in an asymmetric manner. Finally, remyelination completed in the optic nerve facilitated fine visual function recovery in adult zebrafish.

A consensus that newly generated oligodendrocytes contribute to remyelination is widely accepted (Franklin et al. 2020). For example, in vivo imaging from zebrafish larvae showed that olig2^+^ cells could migrate and proliferate after nearby OPCs were specifically ablated in the spinal cord (Kirby et al. 2006) and mouse results showed that NG2^+^ OPCs could proliferate and maintain the balance of OPC numbers in the cortex (Hughes et al. 2013). However, the latest evidence from animal models and human patients has reinvigorated the theory that mOLs mainly contribute to remyelinating but are minimal sources of OPCs (Yeung et al. 2019). However, mOLs have weak capacities to migrate and proliferate due to their complex morphology. The hypothesis that mOLs produced new cells during the remyelination process has remained at the margins of mainstream remyelination neuroscience (Franklin et al. 2020). Evidence in support of this hypothesis has been very rare. This phenomenon in which mOL cells proliferate with the myelin sheath contact was first observed in 1984 (Arenella and Herndon 1984; Ludwin 1984). Then, rare examples of myelinated Schwann cells incorporating ^3^H-thymidine were reported in 1987 (Griffin et al. 1987). Another in vitro study showed that mOL could re-enter the cell cycle when stimulated by growth factors (Fressinaud et al. 1993). However, direct evidence of mOL division, especially direct evidence from in vivo imaging, is still missing. In our results, in vivo imaging of dividing mOLs within the optic nerve and retina strongly supports the idea that mOLs participate in the progression of remyelination (Fig. 3, Fig. 4). However, as zebrafish are lower vertebrate animals, our results may not represent the same phenomenon that may exist in humans and other mammalian species. This possibility aroused our attention because of mOL proliferation during remyelination.

Asymmetric division, a famous classic example, is radial stem cell division in the ventricular zone during development, usually in stem cells. Adult zebrafish, a perfect tissue organ regeneration model, exhibited two examples of asymmetric division after injury. The first example is that Müller glia plays the role of somatic retinal stem cells to produce most kinds of retinal cells after injury. In the normal retina, the Müller glial nucleus is located in the inner nuclear layer, and their processes span the apical-basal extent of the retina. Under injured conditions, the nucleus undergoes interkinetic nuclear migration and then produces neuronal progenitor cells at the outer nuclear layer through an asymmetric pathway (Lahne and Hyde 2017). The second example is that in the transverse sections of 15-dpf Tg (olig2:egfp) fish, anti-phospho-histone H3 (pH 3) antibody labeling revealed an asymmetrical division of olig2^+^ radial glial cell, in which chromosomes were aligned perpendicular to the central canal. The cell that maintains contact with the central canal remains a radial glial precursor, while the other enters the oligodendrocyte lineage (Park et al. 2007). In our in vivo imaging results, oligodendrocyte division is not like OPC division in the development of larval zebrafish, which retracts its processes and rounds up to produce two daughter OPCs in the anteroposterior axis (Kirby et al. 2006). Analysis of the displacement and speed characteristics of the “mother cell” and “daughter cell” shows that the “daughter cell” immediately migrated out of the territory of the “mother cell” at a speed of 1.5984 ± 0.7716 μm/min (*n* = 3) in the first half-hour after division. These “mother cells” only slightly shook at the original position (Movie S2). mOLs (the myelin process maintained integrity, as shown in Fig. 4) divided and budded from the soma, indicating asymmetrical division (Fig. 3 and 4a; Fig. S2; Movies S1, S2 and S3). In the future, novel and recently developed methods (such as optical coherence tomography imaging) will help confirm whether newly generated cells could mature into myelinated oligodendrocytes over a long period of time (Huckenpahler et al. 2020).

Which cell contributes more to visual function recovery? In the LPC-induced myelination model, as all oligodendrocytes in the central area of the optic nerve were killed, OPCs from the adjacent regions should be the only source of remyelination. However, some studies have indicated that OPCs are very fragile, while mOLs are more tolerant of inflammatory environments (Franklin and Ffrench-Constant 2008; Scolding et al. 1998). Crushed optic nerves in goldfish showed that surviving oligodendrocytes are essential for regrowing the myelin sheath to wrapped regenerated axons (Ankerhold and Stuermer 1999). Here, we found that although the “mother cells” of mOLs have a complex morphology that restricts their migration ability, newborn “daughter cells” have a strong ability to migrate (Fig. 4). The mean time for new OPCs to be dissected from the mother cell (307.7 s, n = 3) was similar to the result from larval zebrafish, which only required several minutes for division (Lust and Wittbrodt 2018), and the mean speed of OPC migration was also similar (Kirby et al. 2006) (the mean speed was 1.5984 μm/min, n = 3). This provides the possibility that once new OPCs are born from the mother cells, it is very difficult to distinguish whether they came from pre-existing OPCs or mOLs. This means that the important role of mOLs during remyelination may have been ignored previously (Dimou et al. 2008; Young et al. 2013). We believe that the processes of the differentiation of OPCs, the survival of mOLs, and the proliferation of mOLs have benefited visual function recovery.

Which signaling pathway stimulates mOL cells to reprogram is not clear. In this study, we injected TNFα to promote mOL division (Arnett et al. 2001; Cunha et al. 2020) (Fig. 4). As a proinflammatory molecule released by dying neurons and immune cells in CNS damage paradigms, TNF-α is required for inducing Müller glia to re-enter the cell cycle. Knockdown of TNF-α expression significantly reduced the number of proliferating Müller glia in the zebrafish retinal regeneration response (Nelson et al. 2013). Recent research has reported that the addition of TNF-α strikingly increased newly generated premyelinating and myelinating oligodendrocytes in slice cultures or the cortex of mice (Cunha et al. 2020). P-erk1/2 is a critical protein for the survival and proliferation of oligodendrocytes during remyelination progress (Hu et al. 2008; Lin et al. 2013; Napoli et al. 2012) **a**nd was highly activated at 5 dpi in olig2^+^ cells at the distal stump (Fig. 1f). Activated erk1/2 in pre-existing oligodendrocytes of adult mice could push mOLs to reinitiate myelination by the addition of newly formed myelin wraps to existing myelin sheaths (Jeffries et al. 2016) and increase OPC transient hyperproliferation and overproduction during early development (Ishii et al. 2013). Furthermore, activation of erk1/2 in myelinated Schwann cells was sufficient to increase Schwann cell proliferation in the absence of axonal damage (Napoli et al. 2012). Some results showed that *olig2,* an essential transcript for oligodendrocyte differentiation (Yu et al. 2013), was activated by erk1/2 phosphorylation. In the territory of iPS, the activation of *olig2*, *Sox10,* and *Zfp536* was sufficient to induce fibroblast cells to convert to oligodendrocyte progenitor cells (Yang et al. 2013). Sustained overexpression of *olig2* could be an efficient way to increase OPC generation in the spinal cords of transgenic mice and promote myelin repair in a lysophosphatidylcholine-induced demyelination model (Wegener et al. 2015). Interestingly, olig2:eGFP was not expressed in the normal Müller cells of the adult zebrafish retinal but was expressed in these Müller cells that re-enter the cell cycle after retinal injury (Fimbel et al. 2007). In some diseases, continuous activation of the *olig2* gene is always related to oligodendrocyte hyperplasia (Hughes et al. 2013). In multiple sclerosis lesions, *olig2* displays a differential expression pattern, which prevails in maturing oligodendrocytes at the active lesion borders, rather than chronic silent and shadow plaques (Wegener et al. 2015). As the olig2 gene is continuously expressed in mOLs (Fancy et al. 2014; Lau et al. 2012), it is possible that these cells may have the ability to proliferate under inflammatory conditions (Cunha et al. 2020). However, we have no evidence to contend that mOLs could also produce OPCs under normal conditions.

Zebrafish is a perfect model of vertebrates to image remyelination in vivo (Hu et al. 2018). The larval zebrafish can restore the normal length and thickness of myelin sheaths during cuprizone-mediated demyelination (Karttunen et al. 2017; Powers et al. 2012). We compared the regenerated axon diameter at 6 wpi with the normal axon diameter, and no significant difference was found (Fig. S4A). Although we did not obtain myelin length results directly, we analyzed the density of Ranvier nodes along the regenerated axons (Craner et al. 2005) and found that they had recovered to normal levels from 3 wpi (Fig. 5l). Considering that the myelinated axon density at 6 wpi was slightly higher than that in normal fish (Fig. S4B), we could not conclude that the myelin length in the regenerated optic nerve was shorter than that in normal fish. Interestingly, TEM results showed that remyelination began at 7 dpi (Fig. 1k), while nav1.6 showed linear scattering along the denuded axons (Fig. 5g). This phenomenon suggests that nav1.6 clustering in the regenerated axon of the zebrafish optic nerve was driven by myelin formation, similar to the results in mammals (Waxman 2006). Last, as denuded axons only induced coarse function recovery, fine visual function recovery reflected that remyelination was completed in adult zebrafish (Orger et al. 2008).

## Conclusions

Zebrafish is a perfect model of image remyelination in vivo. Our in vivo imaging results directly revealed that new OPCs are asymmetrically divided from the soma of local mOLs in the optic nerve and within the retina. These finding will highlight the role of mOLs in the progression of remyelination in the mammalian CNS.

## Methods

1. **Zebrafish Lines**

Adult zebrafish 1 ~ 1.5 years of age with a body length between 3.0 ~ 3.4 cm were used. AB/WT and Tg (olig2: eGFP) transgenic lines (Park et al. 2007) were used for different aims. In general experiments, zebrafish were maintained at 28.5 °C with a 14/10 h light-dark cycle and were fed 2 times/day. All surgeries were performed after zebrafish were anesthetized with 0.03% tricaine methane-sulfonate (MS-222, Sigma), and all efforts were made to minimize suffering.

2. **Optic nerve injury**

Optic nerve crushing was described previously (Zou et al. 2013). Briefly, after anesthetizing zebrafish in 0.03% MS-222 solution and exposing the optic nerve of each animal’s left eye, we crushed the optic nerve at the middle site with #5 jewelry forceps (Fine Science Tools) for approximately 10 s. Then, we replaced the eye into its orbit and woke up the zebrafish in the system rearing water. The right eye was kept intact to serve as an internal control.

3. **Optic nerve transplantation**

To image single oligodendrocytes in the optic nerve in vivo, we transplanted optic nerve segments from Tg (olig2:eGFP) (nearly 1.5 years of age) into AB/WT fish (Tian et al. 2016). Before optic nerve transplantation surgery, the host fish (AB/WT line) were raised at low temperature (20 °C) for one week. Then, we anesthetized the donor and host fish simultaneously by cutting the middle segments of the host optic nerve (Vannas Spring Scissors, HF. S. TH) and the donor optic nerve to pave it into the gap of the optic stump in the host fish. The host eye was gently returned into its orbit, and the donor and host optic nerves were tightly contacted. We woke up and maintained the zebrafish in a 20 °C environment and fed them two times per day. Nevertheless, we screened those fish that maintained olig2:eGFP fluorescence one week later for subsequent in vivo imaging.

4. **In vivo imaging of the optic nerve and retina in adult zebrafish**

Although directly pulling the eyeball out of orbit (Koch et al. 2011) is also possible in adult zebrafish, we adopted a more ingenious method to expose the optic nerve while keeping it under normal physiological conditions (Fig. S3A). Briefly, after anesthesia with MS-222, 0.1 μl of α-bungarotoxin (2133, Tocris Bioscience) at 1 mg/ml was injected (Picospritzer III, Parker, USA) into the right ophthalmic vein. We exposed the optic nerve and ophthalmic artery of the right eyeball, cut all six muscles and the optic nerve near the optic chiasm, burned the ophthalmic artery and vein with a heated metal needle, and cleaned the orbit with a piece of gelfoam. Once the bleeding stopped, the Trabeculae Cranii (http://zfatlas.psu.edu/reference.php#ref-100) was removed and the optic nerve of the contralateral eye was exposed. For better observation, the ciliary nerve near the left eyeball was slightly draw and the optic nerve was horizontally placed. For retina in vivo imaging, the cornea and lens were removed, a spitball was padded under the dorsal sclera to lift the retina up, and cover a coverslip was used to flatten the retina (Fig. S3B).

To keep fish alive during long-term imaging, adult zebrafish were embedded in 1.5% agarose in a plastic holding device. A small tube was inserted into each fish’s mouth, and constant well-aerated water (0.01% MS-222) was run through its gills by a gravity drive. Effluent solutions dropped down into a tank under the agarose chamber and were aspirated away by a vacuum tube (Fig. S3C). After placing this plastic holding device under the 40× objective lens (NA = 0.8) of a BX61 microscope (Olympus, Japan), we scanned the in vivo images using FV 1000 software (Olympus, Japan) (Fig. S3D).

5. **Behavior test**

All behavior experiments were conducted in the afternoon between 2 PM and 6 PM. To investigate the fine visual function recovery of ocellus injury, we constructed an optokinetic response (OKR) of ocellanae in adult zebrafish (Zou et al. 2010). As the experimental eye was stimulated by light grating, while the control eye was shielded completely (200 degrees from the tail to the left nasal). The control eye’s nystagmus was driven by the synergistic mechanism of the binoculus reflecting the visual function of the experimental eye. We recorded eye nystagmus both clockwise and counterclockwise when the grating cycle was 12 and the velocity was 25 RPM (CANON A640, 30 frames/s) and analyzed the numbers of nystagmus and the total amplitude frame by frame using our constructed tools.

6. **Drug administration**

To promote the dividing progress of oligodendrocytes in vivo, we injected 60 nl of recombinant cotton rat TNFα (1011-CR-025/CF, R&D, 5 μg/ml) into the eyeball every 4 h (Cunha et al. 2020). To label oligodendrocyte proliferation after TNFα promotion, BrdU (B500, Sigma) was intraperitoneally injected every 4 h at a dose of 200 mg/kg at a concentration of 20 mg/ml diluted in DMSO (D5879, Sigma).

7. **Axon tracing**

For tracing the RGC axon in the optic nerve, a crystal of biocytin (B4261, Sigma) was prepared by soaking small pieces of gelatin foam in biocytin solution after drying it. Briefly, we anesthetized the fish and embedded them in 1.5% agarose as mentioned above, cut the whole optic nerve before the optic chiasma, and immediately positioned biocytin crystals at the stump. After the tracer was absorbed for 2 h, the fish were perfused with 4% paraformaldehyde (pH 7.4, 1× PBS). Biocytin was detected with streptavidin-Cy3 (S6402, Sigma), and images were taken under a BX61 microscope (Olympus, Japan) with FV 1000 software (Olympus, Japan).

8. **Immunohistochemistry**

After anesthesia in 0.03% MS-222, the fish were intracardially perfused with 4% paraformaldehyde (pH 7.4, 1× PBS) for 2 min at a speed of 1 ml/min (Zou et al. 2013) and then fixed in 4% paraformaldehyde at 4 °C overnight. After dehydration in 30% sucrose, 5-μm cryosection slices were produced. Except for BrdU retrieval with 2 N HCl, the general steps are as follows: Cryosection slices were rehydrated three times (5 min/time) with 1× PBS, blocked in 10% PBST (10% serum, 1% BSA, 0.3% Triton X100 in 1× PBS), incubated with the first antibody overnight at 4 °C, washed three times (5 min/time) with 1× PBS, incubated with second antibody 2 h at room temperature, washed with 1× PBS again and mounted in 80% glycerol for imaging.

Mouse anti-BrdU (B2531, Sigma) for detecting newborn cells and mouse anti-Nav1.6 (S8809, Sigma) specifically labeling Ranvier nodes (Voas et al. 2007) were used at a 1:1000 dilution. The custom-made rabbit anti-MBP antibody (1:1000, Abmart) was mentioned previously (Zou et al. 2013). All secondary antibodies were from Invitrogen and were diluted 1:400, including goat anti-mouse Alexa Fluor 488, goat anti-mouse Alexa Fluor 568, donkey anti-mouse Alexa Fluor 647, donkey anti-rabbit Alexa Fluor 488, and goat anti-rabbit Alexa Fluor 635.

9. **Western blot**

Each 10 optic nerves were lysed in 50 μl of SDS sample buffer (Beyotime), supplemented with an equal volume of 2× SDS-page loading buffer, and boiled for approximately 10 min. After centrifugation at 12,000 RPM at 4 °C, 10-μl samples were separated by 12% SDS–polyacrylamide gel electrophoresis, and the protein was transferred onto a polyvinylidene difluoride membrane (Millipore) for antibody recognition. Anti t-erk1/2 and anti p-erk1/2 were both from Cell Signaling and were diluted 1:1000. Horseradish peroxidase-conjugated donkey or rabbit secondary antibodies were used at a 1:10,000 dilution (Sango, China). The proteins were visualized with an ECL detection kit (Sango, China) and imaged via film slicing.

10. **Transmission electron microscopy and immunoelectron microscopy**

Animals were perfused with 4% PFA, and the optic nerve was removed into primary fixative (2.5% glutaraldehyde in 0.1 M phosphate buffer, pH 7.4) overnight at 4 °C. The next steps were followed by standard steps for electron microscopy. Three random fields of the distal stump of the transverse section were imaged by transmission electron microscopy (TEM, JEM-1230, Japan) at 25,000× magnification. To analyze the myelin index, all axons were numbered, and half of the axons were selected randomly. After measuring the inner and outer diameter at three different orientations of each axon, the myelin index was used as the quotient of the inner diameter divided by the outer diameter. The myelin index of the naked axons is 1.

In postembedding immune electron microscopy, ultrathin sections were mounted on a nickel screen. Then, the immunohistochemistry steps were performed as described above. The primary anti-GFP antibody was diluted at 1:50 (2956, Abmart, China) and was incubated with the sections at 4 °C overnight. A second donkey anti-mouse antibody conjugated with 10 nm aerosol (Boster, China) was diluted 1:20 and incubated with the sections at 37 °C for 30 min. Then conventional electron microscopy was performed.

### Statistical analysis

The data were analyzed using one-way ANOVA (by Tukey’s test) in GraphPad Prism version 4.0 (Prism, USA), and the results are shown as the mean ± SEM. The criterion of significance was set at *P* = 0.05. *, ** and *** represent *P* < 0.05, *P* < 0.01 and *P* < 0.001 respectively.

## Supplementary Information

**Additional file 1: Figure S1**. Olig2+ cells rewrapped RGC axons in the host fish. (A-C) Retrograde labeling of the RGC axon from the chiasm stump showing that the axons (red) were ensheathed by olig2^+^ processes (green) in longitudinal slices. (D) Immunoelectron microscopy showing that olig2^+^ processes wrapped axons in normal olig2:eGFP transgenic fish (arrows). (E) An olig2^+^ process wrapped axon in the host fish was also found (arrows). Scale bar: 5 μm (A-C); 100 nm (D-E).

**Additional file 2: Figure S2**. Another case of local oligodendrocyte cells divided in the transplanted optic nerve. As shown by imaging, olig2^+^ cells also have complex morphology with all their processes parallel to the optic nerve. In this case, the progress of cell division is clearly shown. As a transition station, the arrow at the seventh frame indicates concave cytoplasm. Each image has 23 slices, the interval of the Z stack is 1 μm, and the scale bar is 10 μm.

**Additional file 3: Figure S3**. Equipment setup for in vivo imaging. (A) The optic nerve of the left eyeball was exposed from the right orbit. After removing the right eyeball and the connective tissues and the trabeculae cranii, the optic nerve of the left eyeball was exposed. (B) After removing the cornea and lens, the retina was lifted up by padding a spitball under the dorsal sclera. Then, a coverslip was placed on this uplifted retina; this retinal area, indicated by curved dots, was flattened. The imaging fields were acquired from this flattened area, indicated by the rectangular areas.(C) Detail of the imaging equipment. Each fish was embedded in an agarose chamber. Well-aerated solution was inflowed into the mouth by gravity, and after crossing the gills, it dropped into a tank under the agarose chamber and was aspirated by a vacuum tube. A water object lens was located above the coverslip, which was tightly stuck to the orbit (optic nerve imaging) or the retina (retina imaging). (D) A complete view of the in vivo imaging equipment. A three-limb tube connects the MS-222, system water, and the imaging equipment. Time lapse imaging was captured by the FV1000. Scale bar: 200 μm (A, B).

**Additional file 4: Figure S4**. Axon diameter did not contribute to myelin sheath thinning. (A) Axon diameter was not decreased in the regenerated optic nerve at 6 wpi. (B) Myelinated axon numbers at 6 wpi were slightly increased compared with normal fish, but there was no significant change. (C) Scatter diagram of the myelin index and axon diameter at all times after the optic nerve was injured. Except for axons at 1 wpi (mostly axons were smaller than 200 μm), axon diameters within 200–550 μm in all regenerated axons were approximately 74.43% (*n* = 2730), which was similar to normal fish (86.47%, *n* = 303).

**Additional file 5 Movie S1:** The images of the left optic nerve are from the orbit of the right eye; the orientation of the eyeball is low, and the chiasm is up. Benefiting from optic nerve transplantation, olig2^+^ cells scattered along the whole optic nerve in the host. In this video, two olig2^+^ cells project their processes to large areas, suggesting that they were complex oligodendrocytes at 3 mpt. Each image has 25 optic slices, the interval is 1 μm, and the scale bar is 10 μm. Images were collected every 1 min, and the movie runs at 3 frames per second.

**Additional file 6 Movie S2:** This olig2^+^ cell is also a complex oligodendrocyte with all of its processes parallel to the optic nerve. The eyeball is low in each frame. Each image has 23 optic slices, the interval is 1 μm, and the scale bar is 10 μm. Images were collected every 1 min, and the movie runs at 3 frames per second.

**Additional file 7 Movie S3:** To stimulate mOL division, TNFa solution (5 μg/ml) was injected into the eyeball every 4 h. After 12 h, the cornea and lens were removed, and the retina was covered with TNFa solution. Images were collected every 1 min, and each image has 15 optic slices, with an interval of 1 μm. As the mother cell did not migrate, it was tracked with a solid red ball throughout proliferation. The daughter cell migrated quicklyand was tracked as yellow once it was produced. The movie runs at 5 frames per second. The scale bar is 10 μm.

## Data Availability

All data generated or analyzed during this study are included in this published article and its supplementary information files. Requests for materials should be addressed to the corresponding author: halozsq@ncu.edu.cn

## Abbreviations

BrdU: 5-Bromo-2′-deoxyuridine
DMSO: Dimethyl sulfoxide
dpi: Day postinjury
dpt: Days posttransplantation
ERK 1/2: Extracellular regulated protein kinases 1/2
eGFP: Enhanced green fluorescent protein
mOLs: Mature oligodendrocytes
MS: Multiple sclerosis
MS-222: Tricaine methane-sulfonate
OPCs: Oligodendrocyte progenitor cells
mpt: Months posttransplantation
OKR: Optokinetic response
Olig2: Oligodendrocyte transcription factor 2
pH 3: Phospho-histone H3
RGC: Retinal ganglion cell
TEM: Transmission electron microscope
TNFα: Tumor necrosis factor alpha
wpi: Weeks postinjury
WT: Wild-type
