# Supplementary figures and images for: In vivo imaging reveals mature Oligodendrocyte division in adult Zebrafish
Source: Cell Regen. 2021 Jun 2;10:16. doi: 10.1186/s13619-021-00079-3 (PMC8169745; doi:10.1186/s13619-021-00079-3)

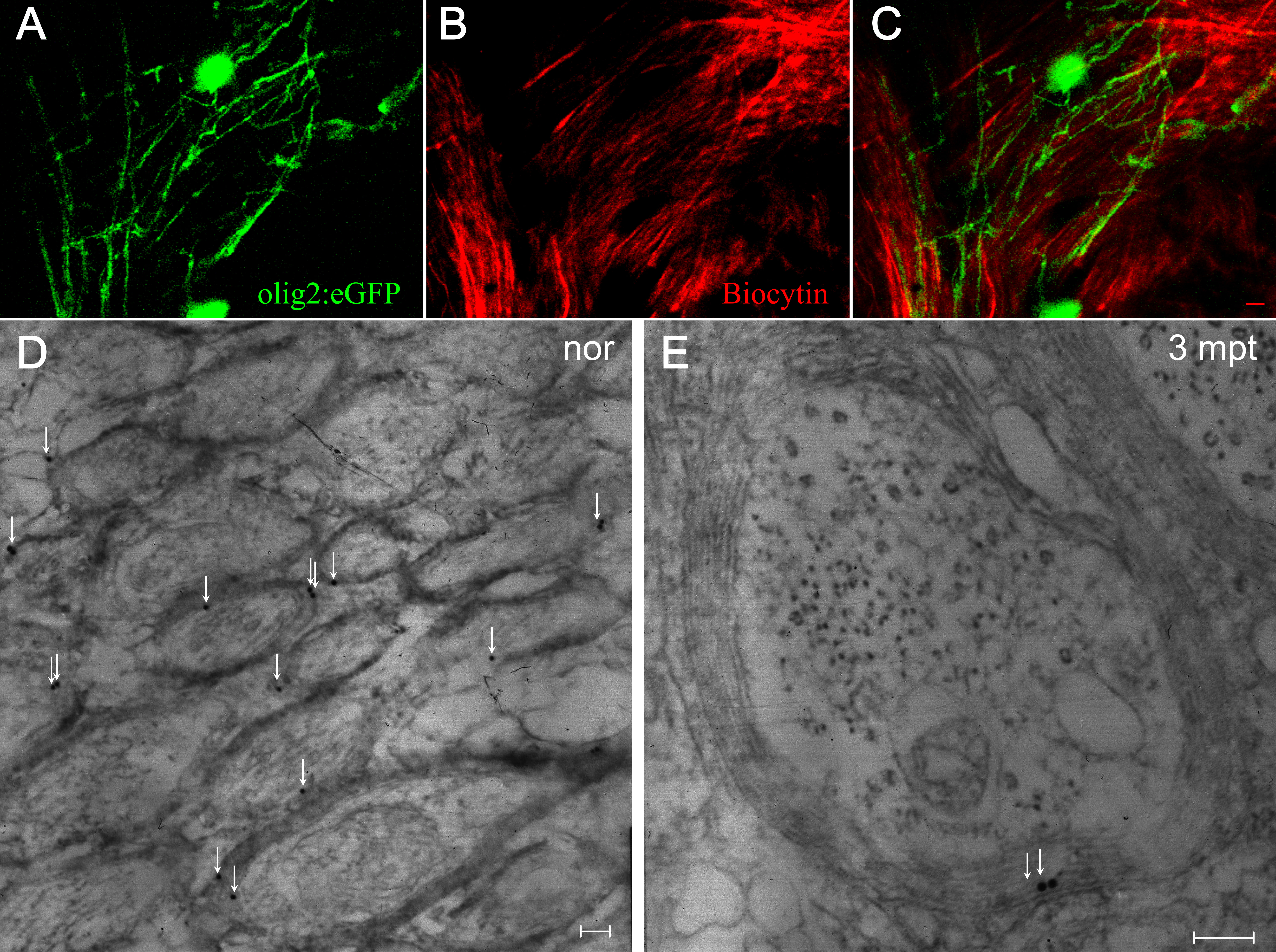

Supplement: Supplementary file 1 — Additional file 1: Figure S1. Olig2+ cells rewrapped RGC axons in the host fish. (A-C) Retrograde labeling of the RGC axon from the chiasm stump showing that the axons (red) were ensheathed by olig2+ processes (green) in longitudinal slices. (D) Immunoelectron microscopy showing that olig2+ processes wrapped axons in normal olig2:eGFP transgenic fish (arrows). (E) An olig2+ process wrapped axon in the host fish was also found (arrows). Scale bar: 5 μm (A-C); 100 nm (D-E). [file 13619_2021_79_MOESM1_ESM.jpg]

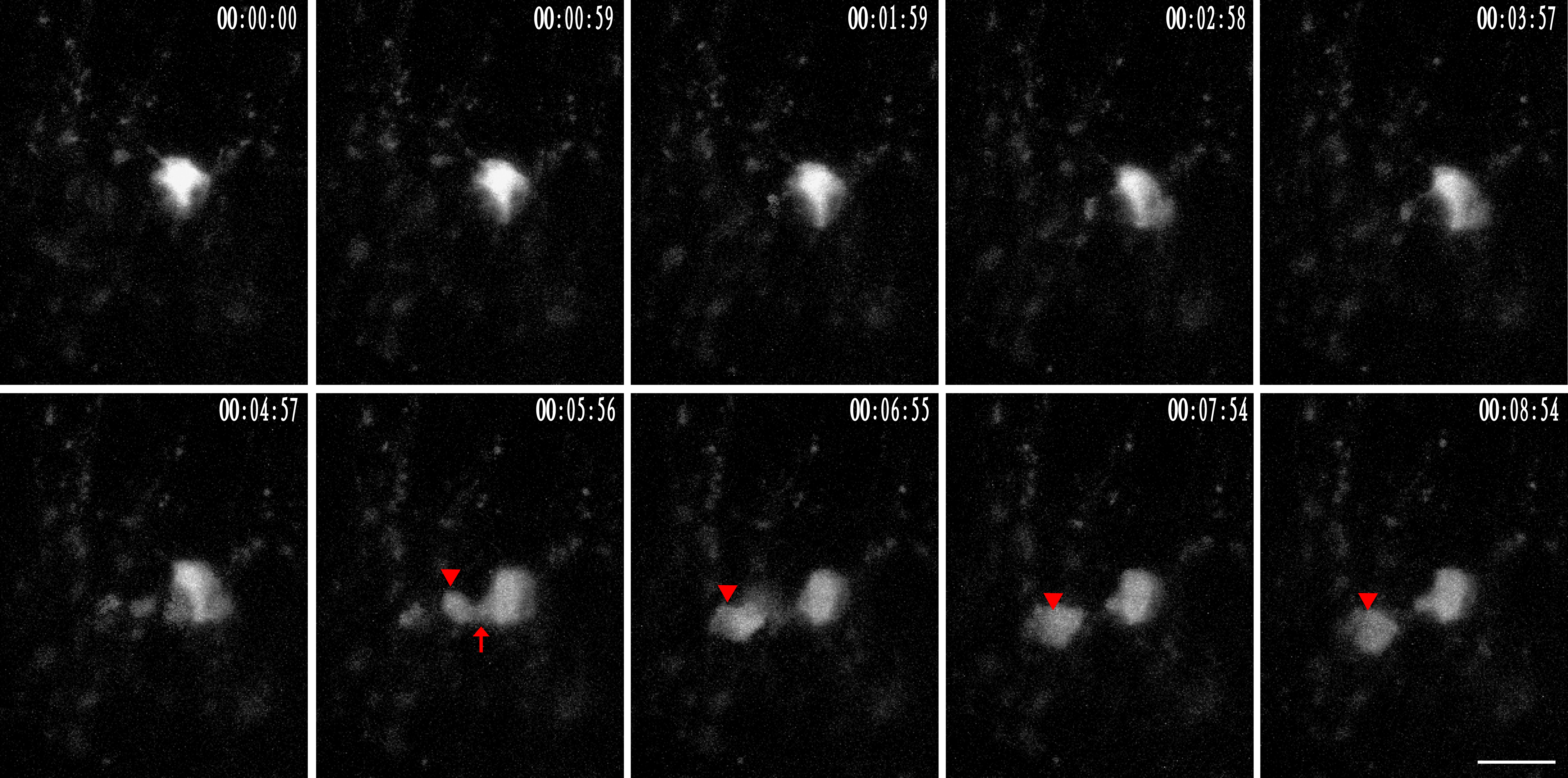

Supplement: Supplementary file 2 — Additional file 2: Figure S2. Another case of local oligodendrocyte cells divided in the transplanted optic nerve. As shown by imaging, olig2+ cells also have complex morphology with all their processes parallel to the optic nerve. In this case, the progress of cell division is clearly shown. As a transition station, the arrow at the seventh frame indicates concave cytoplasm. Each image has 23 slices, the interval of the Z stack is 1 μm, and the scale bar is 10 μm. [file 13619_2021_79_MOESM2_ESM.jpg]

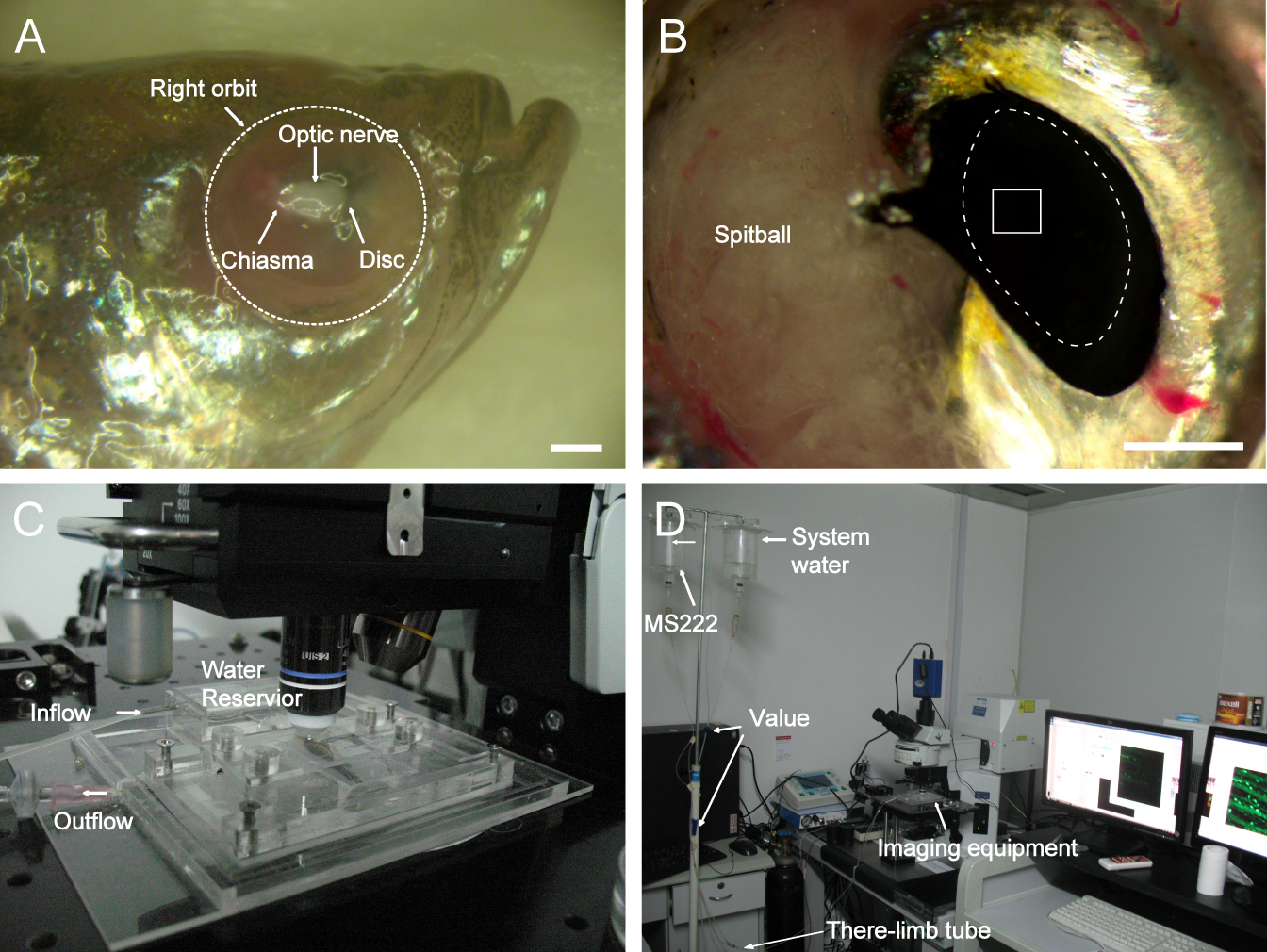

Supplement: Supplementary file 3 — Additional file 3: Figure S3. Equipment setup for in vivo imaging. (A) The optic nerve of the left eyeball was exposed from the right orbit. After removing the right eyeball and the connective tissues and the trabeculae cranii, the optic nerve of the left eyeball was exposed. (B) After removing the cornea and lens, the retina was lifted up by padding a spitball under the dorsal sclera. Then, a coverslip was placed on this uplifted retina; this retinal area, indicated by curved dots, was flattened. The imaging fields were acquired from this flattened area, indicated by the rectangular areas.(C) Detail of the imaging equipment. Each fish was embedded in an agarose chamber. Well-aerated solution was inflowed into the mouth by gravity, and after crossing the gills, it dropped into a tank under the agarose chamber and was aspirated by a vacuum tube. A water object lens was located above the coverslip, which was tightly stuck to the orbit (optic nerve imaging) or the retina (retina imaging). (D) A complete view of the in vivo imaging equipment. A three-limb tube connects the MS-222, system water, and the imaging equipment. Time lapse imaging was captured by the FV1000. Scale bar: 200 μm (A, B). [file 13619_2021_79_MOESM3_ESM.jpg]

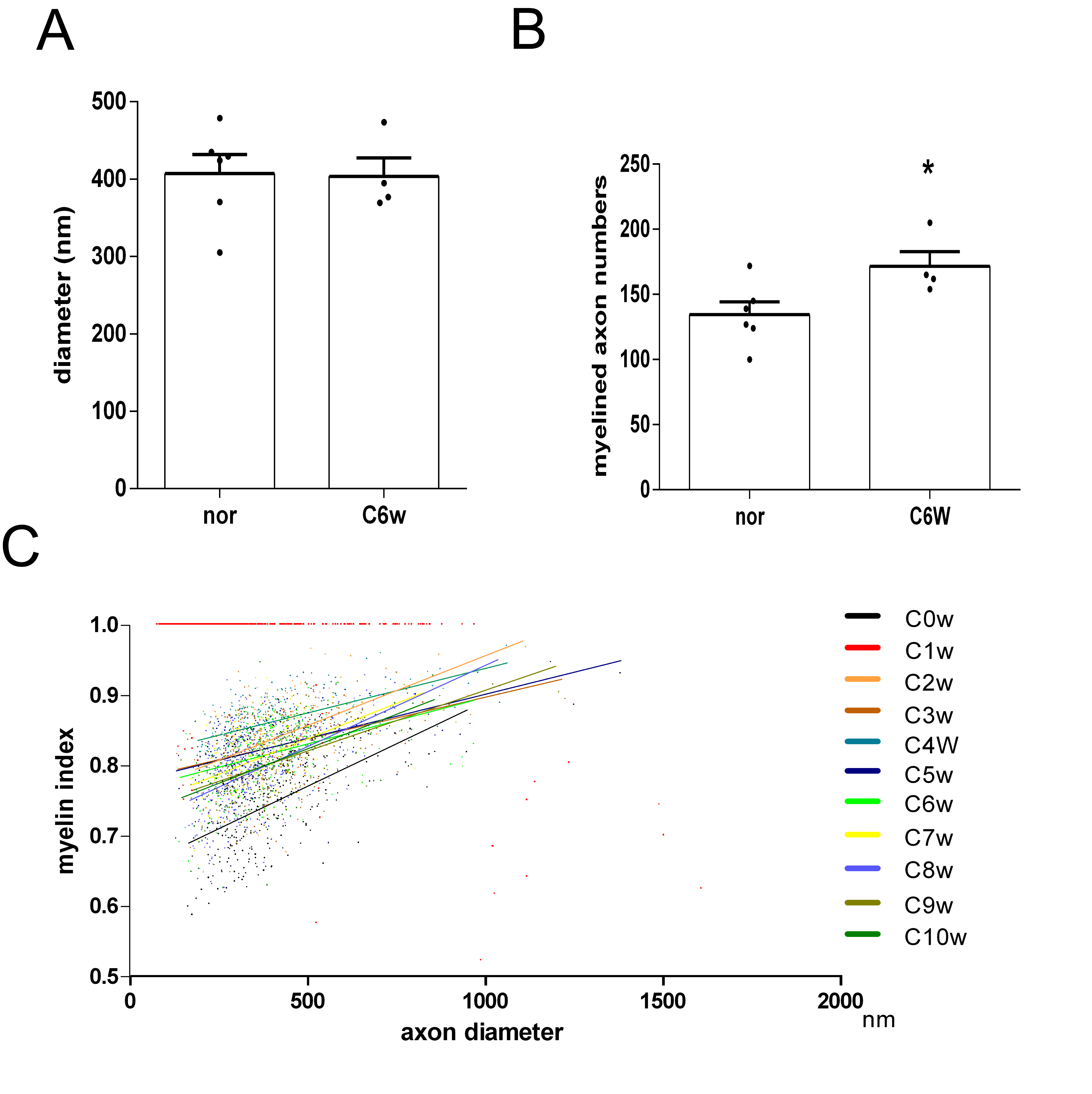

Supplement: Supplementary file 4 — Additional file 4: Figure S4. Axon diameter did not contribute to myelin sheath thinning. (A) Axon diameter was not decreased in the regenerated optic nerve at 6 wpi. (B) Myelinated axon numbers at 6 wpi were slightly increased compared with normal fish, but there was no significant change. (C) Scatter diagram of the myelin index and axon diameter at all times after the optic nerve was injured. Except for axons at 1 wpi (mostly axons were smaller than 200 μm), axon diameters within 200–550 μm in all regenerated axons were approximately 74.43% (n = 2730), which was similar to normal fish (86.47%, n = 303). [file 13619_2021_79_MOESM4_ESM.jpg]
